# Targeted weed management of Palmer amaranth using robotics and deep learning (YOLOv7)

**DOI:** 10.3389/frobt.2024.1441371

**Published:** 2024-10-14

**Authors:** Amlan Balabantaray, Shaswati Behera, CheeTown Liew, Nipuna Chamara, Mandeep Singh, Amit J. Jhala, Santosh Pitla

**Affiliations:** ^1^ Machine Automation and Agricultural Robotics Laboratory, Department of Biological Systems Engineering, University of Nebraska-Lincoln, Lincoln, NE, United States; ^2^ The Jhala Weed Management Laboratory, Department of Agronomy and Horticulture, University of Nebraska-Lincoln, Lincoln, NE, United States

**Keywords:** weed management, robotics and automation in agriculture, machine vision, artificial intelligence, targeted spraying, real time, deep learning, YOLOv7

## Abstract

Effective weed management is a significant challenge in agronomic crops which necessitates innovative solutions to reduce negative environmental impacts and minimize crop damage. Traditional methods often rely on indiscriminate herbicide application, which lacks precision and sustainability. To address this critical need, this study demonstrated an AI-enabled robotic system, Weeding robot, designed for targeted weed management. Palmer amaranth (*Amaranthus palmeri S. Watson*) was selected as it is the most troublesome weed in Nebraska. We developed the full stack (vision, hardware, software, robotic platform, and AI model) for precision spraying using YOLOv7, a state-of-the-art object detection deep learning technique. The Weeding robot achieved an average of 60.4% precision and 62% recall in real-time weed identification and spot spraying with the developed gantry-based sprayer system. The Weeding robot successfully identified Palmer amaranth across diverse growth stages in controlled outdoor conditions. This study demonstrates the potential of AI-enabled robotic systems for targeted weed management, offering a more precise and sustainable alternative to traditional herbicide application methods.

## 1 Introduction

With the anticipated global population increase exceeding 9.7 billion by 2050, the need to increase food production by 102% is becoming increasingly significant (Fukase and Martin, 2020). These unavoidable circumstances arise due to the upcoming challenges of ensuring food security amidst an exploding population, highlighting the criticality of addressing biotic constraints which hamper agricultural productivity. Among these challenges, weeds, pathogens, and animal pests stand out as the major factors exerting significant adverse impacts on crop cultivation across developed and developing nations (Ghersa, 2013). Weeds are a major challenge to agricultural productivity, as they compete with crops for vital resources, creating suitable environments for the growth of harmful insects and pathogens. This dual impact poses a significant threat to crop health and yields (Oerke, 2006). Furthermore, the unchecked spread of weeds disturbs native ecosystems, placing indigenous flora and fauna at risk. This complex relationship between weeds, agricultural systems, and ecological balance underscores the critical need for effective weed management strategies to safeguard agricultural sustainability and biodiversity conservation.

Palmer amaranth (*Amaranthus palmeri* S. Watson), also known as Palmer pigweed or careless weed, is an annual broadleaf weed originating from the deserts in the southwest United States and northern Mexico (Borgato, et al., 2024). This invasive weed has spread rapidly across the United States, significantly impacting various crops, including corn and soybeans, crucial to Nebraska’s agricultural economy. It is ranked as the most troublesome weed in corn, cotton, peanuts, sorghum, and soybean as surveys conducted by the Weed Science Society of America (Van Wychen, 2020; Van Wychen, 2022). Palmer amaranth has unique biological characteristics such as quick growth, higher seed production, wider emergence window, dioecious reproduction, adaptability to broader environmental conditions, and ability to evolve resistance to multiple herbicides, which contributes to its aggressivity, rapid dispersal, and troublesome nature (Borgato, et al., 2024; Ward et al., 2013). It can grow two to three inches daily (USDA-NRCS, 2017). It has an extended germination and emergence period, with several cohorts emerging after planting the main crop, such as corn and soybean (Jha and Norsworthy, 2009). This advantage of multiple cohorts, coupled with its rapid growth, allows the Palmer amaranth to outcompete most crops, leading to yield losses of up to 91% in corn (Massinga et al., 2001).

Conventional herbicide spraying practices frequently encounter inefficiencies due to indiscriminate usage. For example, broadcast applications waste herbicide by spraying at locations where the weeds are not present. Building upon the achievements of conventional herbicide application practices, our methodology underscores the advantages of spot-spraying techniques to address these challenges. The widespread adoption of chemical weed control through herbicides revolutionized agriculture in many regions. This led to the significant reduction of labor costs and dependence on mechanical methods. However, over reliance on herbicides for weed control led to the development of herbicide resistance in weeds. This concern of weeds developing herbicide resistance poses the risk of reverting farmers to tillage practices, and this shift jeopardizes the advancements made in soil conservation through the adoption of no-till farming methods (Clay, 2021). Instead of broadcast application, our approach targets specific areas, where the weeds are detected, maximizing efficacy while minimizing environmental impact. In this study, by focusing on precision application, we aim to optimize herbicide use, reduce wastage, and mitigate off-target effects. Through this refined strategy, we seek to enhance weed control practices, promoting sustainable agricultural systems that balance efficacy with environmental protection. Research has demonstrated that spot spraying technology can reduce herbicide volume usage by 20%–60% compared to broadcast application methods (Ball and Bennett, 2008; Fischer et al., 2020; Young et al., 2008). The spot-spraying techniques have the potential to slow down the development of herbicide-resistance in weeds (Genna et al., 2021). The augmentation of precise weed mapping and species identification methodologies has enhanced the ability to identify and manage invasive species more effectively, promoting enhanced biodiversity and ecosystem health. Moreover, this strategic application increases farmer productivity while mitigating human and environmental exposure to harmful chemicals.

Agricultural robots show potential in implementing intelligent weed management strategies, which could lead to improved efficiencies and higher crop yields. This potential has encouraged the application of robotics and Artificial Intelligence to accurately identify and control weeds (Upadhyay et al., 2024b). A recent study by Upadhyay et al. (2024a) demonstrated a smart sprayer system for site specific weed management in row crop agriculture. The designed system features three nozzles, each separated by 20 inches with an approximate overlap of 50%. The system works by activating a specific nozzle when it detects a weed within its 20-inch coverage area. Another similar study by Partel et al. (2019) demonstrated a low-cost smart sprayer technology which leverages the power of Artificial Intelligence to perform precision weed management. Researchers and the farming community are excited about the possibilities these technologies offer for more precise and sustainable agricultural practices specifically for site specific weed management.

In summary, the main contributions of this study were to:

• Generate a labelled image dataset of Palmer amaranth weed over a window of 45 days post-planting in a simulated corn field for training a weed detection model using YOLOv7.
• Build a one-degree-of-freedom automated gantry sprayer which can precisely spray detected weeds and integrate this gantry system onto a mobile robotic platform.
• Evaluate the developed system in a controlled outdoor environment with greenhouse-grown corn rows and randomized Palmer amaranth in tubs (30 days post-planting).

## 2 Materials and methods

### 2.1 Data collection and dataset preparation

**FIGURE 1:**
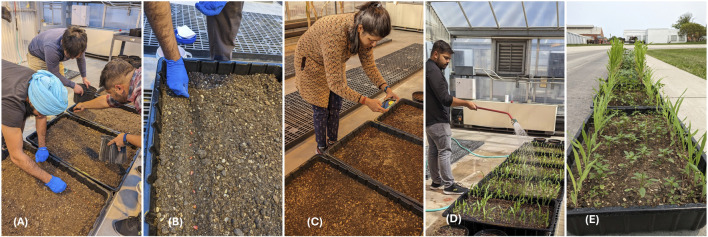
**(A)** Preparing the soil bed for planting corn and Palmer amaranth. **(B)** Sowing the corn in rows and broadcasting palmer amaranth seeds. **(C)** Capturing images of palmer amaranth and corn after germination **(D)** Irrigating the crops **(E)** Tubs arranged on a concrete pavement with two rows of corn and randomized palmer amaranth simulating field-like conditions.

For the purpose of training a deep learning model aimed at targeted spraying of weeds, a comprehensive dataset of Palmer amaranth images was curated. To simulate authentic field conditions, a controlled environment was established within a greenhouse, utilizing five rectangular tubs (610 mm wide, 914 mm long, 305 mm deep) and eighteen pots (305 mm diameter, 305 mm deep), planted with sterilized soil. The tubs were sown with corn in two rows, spaced 381 mm (15 inches) apart (see Figure 1B), while Palmer amaranth seeds were broadcasted across the surface. Out of the eighteen pots, twelve pots contained Palmer amaranth, whereas the other six were planted with corn. Over a span of 45 days, images were systematically captured using a standard smartphone (Model: Pixel 7, Google, Mountain View, California), the images were taken using a range of angles and heights to document the plants’ growth stages. This periodical image collection yielded a dataset comprising a total of 660 photographs obtained from both rectangular tubs and pots (with an original resolution of 2268 × 4032).

The images were meticulously annotated using the “labelImg” tool, resulting in text label files that provided 8,462 labels across two classes: “corn” and “palmer”. The dataset was divided into training, validation, and testing subsets in an 8:1:1 ratio for model training. This stratified sampling ensured a robust and comprehensive dataset, which included various developmental stages of the plants. The resulting dataset and annotations were foundational for developing and validating deep learning models to distinguish corn from Palmer amaranth.

This study demonstrated domain adaptation. With the images captured from the greenhouse setting, the generated dataset may introduce biases when compared to field-grown crops. In the field, weed emergence is often unpredictable, with weeds appearing either in clusters or widely dispersed. Real field conditions were simulated by randomly broadcasting Palmer amaranth seeds. Additionally, images captured in the greenhouse were taken under consistent lighting, whereas outdoor images under natural sunlight vary in illumination, shadow, as well as the plants might sway in the wind, and overall image quality, contributing to potential discrepancies between domains.

### 2.2 Weed detection model

YOLOv7 was one of the best state-of-the-art deep learning models for object detection at the time of the investigation (Narayana and Ramana, 2023; Wang et al., 2022). YOLOv7 is a powerful detector that can detect various objects if the model is trained with a particular image dataset. YOLOv7 achieves a better balance between detection speed and accuracy (Boesch, 2023). In addition, studies have shown YOLOv7 outperforms earlier YOLO versions and other models like Faster R-CNN in weed detection tasks, with higher precision and recall rates (Narayana and Ramana, 2023). This study used the YOLOv7 P5 model to train with our custom weed dataset composed of RGB images. The model identified corn, and the Palmer amaranth weed, generating bounding boxes around them. The detector output consisted of the coordinates for the bounding boxes relative to the image frame, which was then used for targeted spot spraying. The YOLOv7 model was trained in a Dell Precision 7,670 mobile workstation (12th Gen Intel Core i9-12950HX, 32GB RAM, NVidia RTX A4500 16 GB), using PyTorch framework.

### 2.3 Robot platform

**FIGURE 2:**
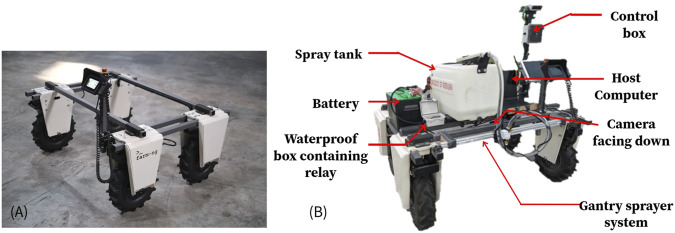
**(A)** The Amiga robot from Farm Ng consists of four hub motors, structural beams, a display, and a controller **(B)** The Amiga robot after it was integrated with the components developed for this research (Weeding robot).

For this study, the Amiga robot platform (Model: Amiga, farm-ng, Watsonville, California) was selected as the deployment platform due to its versatility and suitability for agricultural applications. As shown in Figure 2A, the Amiga is an electric 4-wheel drive skid-steer micro tractor with an NVIDIA Jetson Xavier NX serving as its on-board computing unit (Amiga’s brain), which hosts Robot Operating System (ROS) Noetic (Farm-ng, 2024). It comprises four 250–500 W sealed brushless DC geared hub motors, each motor generating has a peak torque of 140 Nm. It generates a maximum of 2000 W (2.7hp) of motor power. The IP65 waterproof rating on the robot ensures protection against liquid and dust, making it ideal for outdoor farm environments.

The base platform weighs about 145 kg (320 lbs) and is designed to handle a payload of 454 kg (1,000 lbs). Its speed range of 0.18–9.1 km/h accommodates both precise operations and efficient field traversal. Two discrete, hot-swappable 44VDC lithium-ion batteries with a total capacity of 15 Ah were used to power the robot. Using the batteries with full charge, the robot can provide a runtime of between 3 and 8 h, depending on the usage, payload, and terrain (Farm-ng, 2024). High draft applications such as towing, tilling, and soil engaging applications will consume more power and reduce the runtime down to approximately 2–3 h. On the other hand, light-duty applications like hauling materials, data collection, etc., extend the runtime to approximately 6–7 h continuously (Farm-ng, 2024). The Amiga’s integration of robust hardware, advanced computing capabilities, and farm-specific design features provides a comprehensive solution for exploring automated farming techniques in real-world conditions.

### 2.4 Automated gantry sprayer mechanism

**FIGURE 3:**
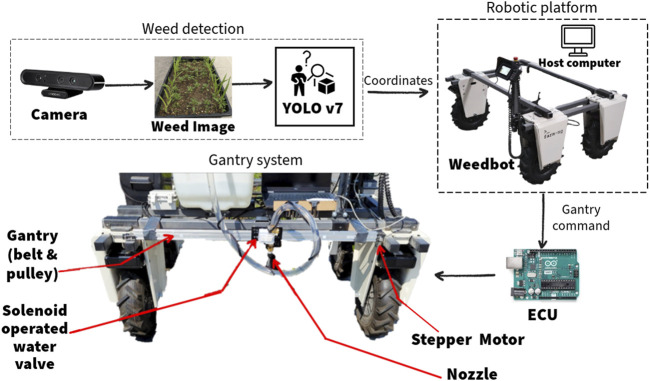
Process flow of the Weeding robot with all the components integrated. The YOLOv7 will generate bounding boxes of palmer and corn from the images captured from the camera and send the coordinates of the detected palmer to the ECU (Arduino UNO) and actuates the stepper motor and the solenoid valve to spray at each of the detected palmer.

An automated single-nozzle sprayer gantry was designed and mounted on the Weeding robot to meet the requirements of this study, as shown in Figure 2B. The sprayer gantry on the Weeding robot consisted of a 1,000 mm long, 20 × 40 mm cross-section T-slot aluminum extrusion profile with extruded linear rail guide, a 6 mm timing belt, a belt straighten tensioner, a 60 × 80 mm gantry plate with six V-type pullies, a NEMA 17 stepper motor with motor mounting plate, an Arduino UNO microcontroller, and a TB6600 stepper motor driver. A 13-gallon liquid tank with a rated 1 GPM (Gallon per minute) pump was installed on the Weeding robot to store and spray the herbicide. The gantry carries a nozzle and solenoid valve to control the spray tank outlet. The Arduino UNO controls the stepper motor for positioning the nozzle and the solenoid valve via a relay to turn the nozzle on and off (see Figure 3). An independent 12 V lead acid battery powered the spraying mechanism and controller. The electronics were enclosed in a weatherproof box.

The microcontroller was connected to the TB6600 stepper motor driver to drive the stepper motor. The microcontroller accepted instructions from the host computer as serial data via USB connection and moved the nozzle on the gantry accordingly. To move the nozzle on the gantry, the required number of stepper motor steps to reach each position was calculated based on Equation 1:

$$n=\frac{xN}{W}$$ (1)

n = no. of the stepper motor steps to the weed location,

N = Total no. of the stepper motor revolution for the full span of the gantry,

W = width of the image (pixels),

and x = X coordinate of detected weed in the image (pixels).

In this experiment, the gantry movement was soft constrained using the boundary width of the camera view, approximately 910 mm, as the sprayer should not spray the location where the camera cannot see.

### 2.5 Host computer

The main computer was a Dell Precision 7,670 mobile workstation (12th Gen Intel Core i9-12950HX, 32GB RAM, NVidia RTX A4500 16 GB). This computer was computationally more powerful than the built-in NVIDIA Jetson Xavier NX on the Amiga robot platform used in this study. The same computer was used to train and host the deep learning models in real-time and host the ROS to communicate with Weeding robot’s computing hardware to control the navigation of the robot and the gantry sprayer that was developed.

### 2.6 ROS bridge

The Weeding robot was developed to utilize gRPC services for communication and control (gRPC, 2024; Farm-ng Developers, 2024). To enable direct control of the Weeding robot using an external computer via ROS commands, a ROS bridge was established to connect it to the Weeding robot’s brain (built-in computing unit). Users can communicate and control the Weeding robot’s movement by sending a twist message using the ROS bridge via the custom ROS topics (/amiga_cmd_vel). A twist message is a data structure comprised of linear and angular velocities in the x, y, and *z* axes of the robot’s relative frame of reference.

The ROS bridge, compatible with ROS Noetic, was connected to the robot’s gRPC services and enabled users to publish twist messages via the robot’s drive topic (/amiga_cmd_vel). These messages were interpreted and converted into CAN messages to control each wheel motor accordingly. Currently, there are three approaches to use the amiga_ros_bridge: (1) Operating the amiga_ros_bridge (and ROS master) directly on the Amiga’s brain, while other ROS nodes on the external computer connect to the Amiga ROS master. (2) Operating the amiga_ros_bridge (and ROS master) on the external computer and establishing a connection to the Amiga CANbus service via gRPC. (3) Operating the amiga_ros_bridge (and ROS master) on the external computer, utilizing the mock server (Farm-ng Developers, 2024). For this experiment, the first method, accessing the Weeding robot’s ROS bridge via SSH, was chosen for its simplicity in implementation.

### 2.7 System operations for targeted spraying

The system operation began with accessing the Weeding robot’s terminal via SSH and initiating the ROS master. Subsequently, the main host computer launched the weed detection node and serial communication node. The serial node facilitated communication with the microcontroller, which controlled the gantry movement and the solenoid valve. The camera (Model: Astra S 3D, ORBBEC, Troy, Michigan) captured images and passed the images through the weed detection node, which used a YOLOv7 object detection model, for generating coordinates of detected weeds.

**FIGURE 4:**
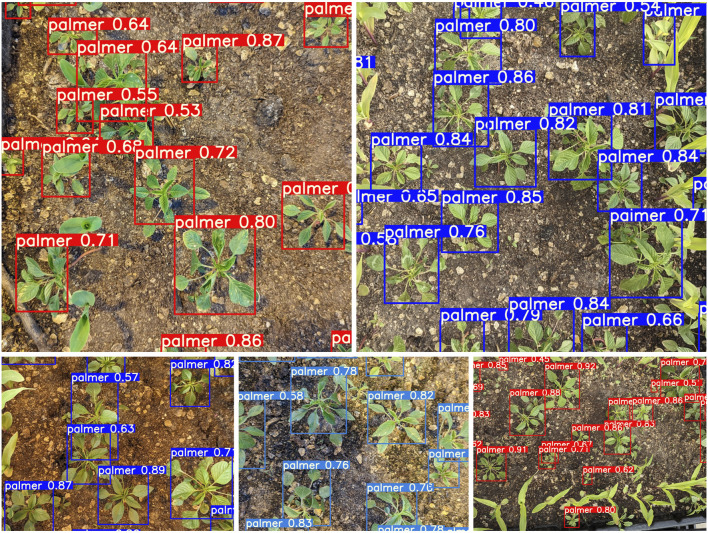
The test result of the YOLOv7 weed detector.

The host computer sent the coordinates of the detected weeds from the weed detector to Arduino via serial communication. The Arduino then triggered the stepper motor which rotated the timing belt thereby moving the gantry head to the targeted position. When the nozzle was precisely above the targeted weed, the Arduino activated a relay to trigger the solenoid valve, resulting in the liquid being sprayed onto the detected weed. Water was used in this study for evaluating the targeted spraying. Throughout this process, the pump remained continuously operating. The pump used was self-priming and can run dry. It can shut off automatically, due to the pressure build-up, when the solenoid valve was shut. After spraying the liquid on all the weeds detected in an image, the gantry head moved back to the start position and the Weeding robot advanced forward. The velocity and direction of the Weeding robot were published on the drive topic (/amiga_cmd_vel). The robot halted to capture another image, and the aforementioned process repeated. Figure 4 presents the process flow of the Weeding robot, integrated with the automated gantry system and weed detection components.

### 2.8 Outdoor experiment

In this paper, two batches of Palmer amaranth weeds and corn plants were cultivated in a greenhouse. While the first batch was used to generate dataset for training the deep learning, a second batch was established using five rectangular tubs for the outdoor experiment. The latter batch featured two rows of corn with 15-inch spacing and randomly broadcasted Palmer amaranth, simulating corn rows environment, specifically prepared for outdoor robotic spot spraying trials. During outdoor trials, the tubs containing 30-day-old plants were moved onto a concrete pavement, and they were aligned in a straight line, extending over a total length of 4.6 m under direct sunlight. This arrangement facilitated semi-realistic conditions for evaluating the Weeding robot’s performance. However, the 30-day-old corn plants exceeded the clearance of the Weeding robot platform, so the corn plants were trimmed to facilitate the maneuverability and ensure unobstructed camera capture.

The decision to cultivate the second batch of crops and weeds in a greenhouse was influenced by the experiment’s timing, which commenced in March, the onset of spring in Nebraska. During this period, farm fields were not yet ready for planting operations, preventing the creation of a testbed in a real agricultural environment with varying conditions. Consequently, the greenhouse provided a controlled setting, and the sterilized soil ensured only plants of interest to be grown to simulate field conditions and ensure the timely progression of the research.

During the test, the rectangular tubs were arranged in a straight line and the Weeding robot was initially positioned at one end of the tubs. The Weeding robot advanced a small interval at a time and captured top-down images at each stop. The gantry was aligned such that the camera’s field of view covered the width of the tub.

### 2.9 Evaluation metric

To evaluate the system performance, mAP (Mean-Average-Precision) was used to evaluate the palmer detection YOLOv7 model. Precision is the ratio of true positive (correct prediction) to overall positive (sum of all detection) (Equation 2) whereas recall is the ratio of true positive to all the truth labels (true positive and false negative) (Equation 3). An AP (Average-Precision) is the average precision value from varying confidence threshold of the detector. A mAP is the mean value of the AP across all classes. In short, the mAP is shown as below (Equation 4):

$$\mathrm{Precision}=\frac{\mathrm{TP}}{\mathrm{TP}+\mathrm{FP}}$$ (2)

$$Recall=\frac{TP}{TP+FN}$$ (3)

$$mAP=\frac{1}{n}\sum_{k=1}^{n}AP_{k}$$ (4)

## 3 Results and discussion

### 3.1 Model evaluation using test dataset

**FIGURE 5:**
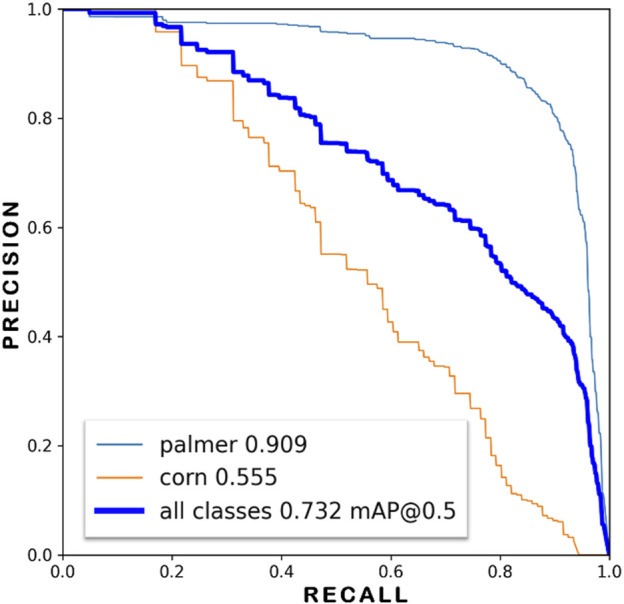
Precision-recall curve of the YOLOv7 weed detection model.

**FIGURE 6:**
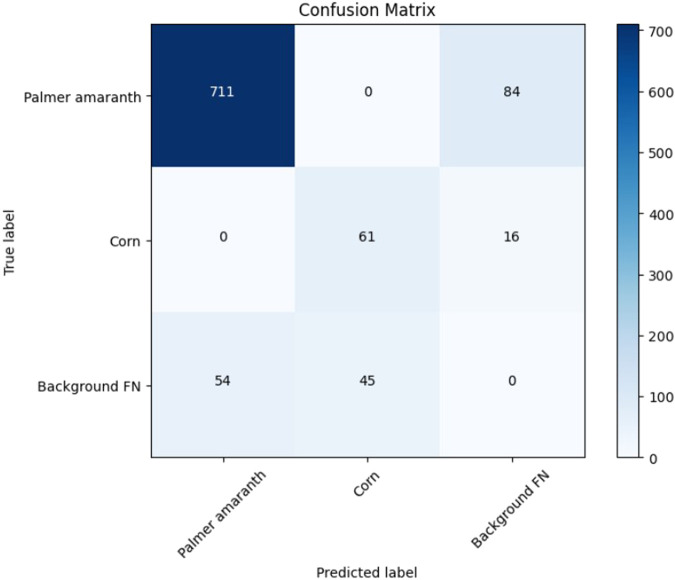
Confusion matrices from the YOLOv7 model test.

The YOLOv7 model was trained with the weed dataset using 320 × 320 input resolution and over 200 epochs. When evaluated against the test dataset of 66 images with 765 labels of palmer and 106 labels of corn which was developed during the dataset preparation step, as mentioned in section 2.1 with a 50% IoU threshold, the overall mAP score reached 73.2%, with AP for Palmer amaranth and corn classes at 90.9% and 55.5%, respectively. A collage of the test results is shown in Figure 4. Figure 5 shows the precision-recall curve with mAP scores of the YOLOv7 weed detector. The model correctly identified 93% of the actual palmer (weed) as palmer while only 58% of the corn as corn. This is due to the dataset being imbalanced, with most of the labels being palmer. However, the success in detecting palmer was considered more critical in this experiment as the sprayer should not spray at the corn. Figure 6 shows the confusion matrix of the test result, the model did not misidentify between palmer and corn. This is due to the dataset design including crop and weed, in this case, Corn and Palmer amaranth. By training the YOLOv7 model with the two classes, the model “learned” the difference and was able to distinguish between them. This is a critical feature of this study by preventing the weeding robot from mis-spraying at the crop. Furthermore, the model’s positive detection of corn plants can be improved, by increasing the number of labels of corn plants. Additional images of corn plants will need to be taken and properly labeled.

### 3.2 System evaluation with outdoor trials

To evaluate the detection model together with the spraying mechanism, outdoor trials were conducted. The second batch of weed and corn mentioned in section 2.8 was used to demonstrate the robotic spot spraying operation under real world application. The experiment was repeated three times in which the Weeding robot passed over the same rectangular tubs with corn rows and Palmer amaranth under the same weather (lighting) conditions. The weed detection model and the sprayer gantry’s performance were evaluated using the results from the experiment.

In the best trial, the Weeding robot used 25% confidence level, accurately identified and sprayed 24 out of 37 Palmer amaranth plants, achieving a 64.9% recall rate (see Table 1). In the same trial, the Weeding robot also mis-sprayed on 13 locations without Palmer amaranth, resulting in a 64.9% precision rate. A lower confidence level of 25% was needed due to the difference in the training data and the real-world application, particularly the lighting conditions where it was much brighter during field trials, demonstrating the challenges of domain adaptation.

**TABLE 1:** The outdoor experiment results, note that the TP = correctly sprayed palmer, FN = missed palmer, FP (empty) = incorrectly sprayed empty spot, FP (corn) = incorrectly sprayed corn.

Trial	TP	FN	FP (empty)	FP (corn)	Precision (%)	Recall (%)
1	24	13	13	0	64.9	64.9
2	21	16	14	0	56.8	60.0
3	22	15	14	0	59.5	61.1
				Average	60.4	62.0

**FIGURE 7:**
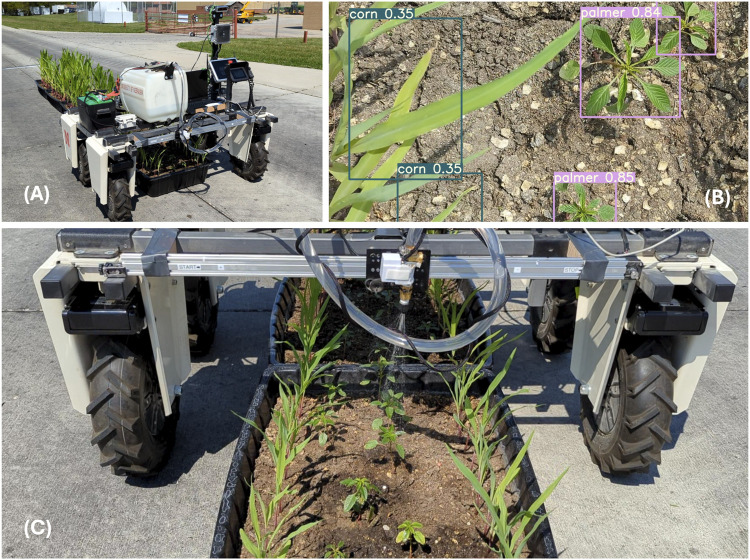
**(A)** The experiment set up of the Weeding robot over the outdoor semi-realistic spot spraying experiment; **(B)** The detection results of the detector; **(C)** the sprayer accurately spraying at the detected weed.

Overall, the Weeding robot achieved an average of 60.4% precision and 62% recall. Notably, the detector did not incorrectly identify corn as palmer and did not incorrectly spray at any of the corn plants. Using a 320 × 320 pixels input size, the detector achieved 50 frames per second during real-time inference using the RTX A4500 laptop graphic card (see Figure 7). Overall, this demonstrated acceptable performance given the model was trained on a small dataset and operated under different environments (greenhouse vs. direct sunlight). While the performance of the weeding robot did not achieve near 100% accuracy, the weeding acuracy can be improved with a larger dataset with more variety of the environment, such as including outdoor weed images at difference time of the day. In addition, a deeper neural network can be used to improve the accuracy at the cost of latency. A 64.9% recall rate indicates 35.1% of Palmer not being sprayed and eliminated, and 64.9% precision indicates 35.1% herbicide were wasted for not spraying at the Palmer. The remaining unsprayed Palmer can negatively impact the crop yield.

## 4 Conclusion and future scope

This research highlights the transformative potential of robotics and deep learning-based models in performing autonomous targeted weed management in agriculture. The dataset developed in this research consisted of 660 labeled images of corn and palmer amaranth sourced from greenhouse-grown crops. Using this dataset, a custom trained YOLOv7 model was trained. It was incorporated into a robotic platform (Weeding robot) with a gantry-based sprayer, making it capable of identifying Palmer amaranth among corn plants and spot spraying the weeds in real-time. This methodology ensured efficient weed targeting and spraying, leveraging advanced deep-learning models for precise detection and robotic automation for targeted herbicide application. The integration of these technologies within the system demonstrated a robust approach to automated agricultural weed management. The system achieved an average of 60.4% precision and 62% recall. The accuracy of the Weeding robot can be substantially enhanced by expanding the dataset with more images of Palmer amaranth, particularly those captured under various field conditions. This would enable the model to learn from a wider range of features, improving its detection capabilities in real-world agricultural environments. Expanding the object detector to identify multiple weed species is another critical area of focus. By improving the system’s ability to distinguish between different types of weeds, we can develop more targeted and effective treatment strategies. Additionally, the gantry-based sprayer system demonstrated a method where a single nozzle can localize itself based on the position of the detected weed and spray the weed. This design minimizes the number of spray nozzles required on the boom, thereby reducing both costs and potential points of failure associated with spray nozzles.

## Data Availability

The raw data supporting the conclusions of this article will be made available by the authors, without undue reservation.
